# Expression of MicroRNAs in the Eyes of Lewis Rats with Experimental Autoimmune Anterior Uveitis

**DOI:** 10.1155/2015/457835

**Published:** 2015-12-02

**Authors:** Yung-Ray Hsu, Shu-Wen Chang, Yu-Cheng Lin, Chang-Hao Yang

**Affiliations:** ^1^Department of Ophthalmology, Far Eastern Memorial Hospital, New Taipei City 22056, Taiwan; ^2^Department of Ophthalmology, National Taiwan University Hospital, Taipei 10002, Taiwan; ^3^National Taiwan University College of Medicine, Taipei 10002, Taiwan

## Abstract

*Purpose.* This study aimed to determine the dynamic changes of NF-*κ*B-related microRNAs (miRNAs) and cytokines over the course of experimental autoimmune anterior uveitis (EAAU) and elucidate the possible immunopathogenesis.* Materials and Methods. *Uveitis was induced in Lewis rats using bovine melanin-associated antigen. The inflammatory activity of the anterior chamber was clinically scored, and leukocytes in the aqueous humor were quantified. RNA was extracted from the iris/ciliary bodies and popliteal lymph nodes to reveal the dynamic changes of eight target miRNAs (miR-155-5p, miR-146a-5p, miR-182-5p, miR-183-5p, miR-147b, miR-21-5p, miR-9-3p, and miR-223-3p) and six cytokine mRNAs (IFN-*γ*, IL-17, IL-12A, IL-1*β*, IL-6, and IL-10).* In situ* hybridization of miRNA and enzyme-linked immunosorbent assay quantification of cytokines were performed to confirm the results.* Results*. Disease activity and leukocyte quantification were maximum at day 15 after immunization. The profiling of miRNA revealed downregulation of miR-146a-5p, miR-155-5p, miR-223-3p, and miR-147b and upregulation of miR-182-5p, miR-183-5p, and miR-9-3p. Cytokine analysis revealed IFN-*γ*, IL-17, IL-12A, IL-1*β*, and IL-6 overexpression, with IL-10 downregulation.* Conclusions.* Dynamic changes of miRNAs were observed over the course of EAAU. By initiating NF-*κ*B signaling, the expressions of downstream cytokines and effector cells from the Th17 and Th1 lineages were sequentially activated, contributing to the disease.

## 1. Introduction

Uveitis is defined as the inflammation of uveal tracts. Because of the heterogeneity of its pathogenesis, recurrent disease attacks, prolonged or repeated steroid treatment is the current mainstay. However, this treatment strategy brings about some problems. Firstly, multiple administrations of steroid might cause subsequent ocular complications, such as cataract, glaucomatous optic neuropathy, scleral melting, or even superimposed infection. Secondly, since no reliable markers can predict upcoming recurrence in preclinical stages, steroid usage only alleviates but not prevents uveitis attacks. Therefore, uveitis still accounts for 10–25% of legal blindness worldwide [1–3]. Among the anatomical classifications by the Standardization of Uveitis Nomenclature (SUN) Working Group [4], 43–70% of uveitis cases are anterior uveitis [5–7]. Despite the well-described clinical presentations, the exact underlying mechanism of the disease has not yet been completely elucidated.

Several animal models have been developed for the further study of uveitis. Among these, as established by Broekhuyse and colleagues, experimental autoimmune anterior uveitis (EAAU) on Lewis rats differs from another common model, experimental autoimmune uveoretinitis (EAU), in that the inflammation remains exclusively anterior, and the photoreceptor cells and retinal tissues are not affected [8]; this resembles human acute anterior uveitis (AAU). Moreover, the clinical course of EAAU is also similar to human AAU. It often exhibits disease onset at day 11 after immunization, with inflammation peaking at days 15–19, recovery at day 30 [9], and it has a recurrent nature [10]. Immunologically, previous literature has revealed the essential involvement of the nuclear factor kappa B (NF-*κ*B) pathway in EAAU, with the subsequent secretion of numerous downstream cytokines and production of chemokines [11, 12]. While innate immunity contributes to both the disease induction and tissue damage, adaptive immunity, particularly Th1/Th17 activation, is regarded as being crucial in some panuveitis studies [13–15]. However, researches on the dynamic involvement of Th1/Th17-related cytokines in EAAU have been inconclusive [11, 16].

MicroRNAs (miRNAs) are small noncoding RNA molecules that can function as posttranscriptional regulators of gene expression and affect numerous biological processes in eukaryotes [17]. Recently, more information on the relationship between miRNA and immunity has been elucidated [18–20]. It has been suggested that the interplay of miRNAs and NF-*κ*B can regulate the immune response either positively or negatively [21, 22]. Specifically, miR-146a and miR-155 are considered as key immunological players. By attenuating tumor necrosis factor (TNF) receptor-associated factor 6 (TRAF6) and interleukin- (IL-) 1 receptor-associated kinase 1 (IRAK1), miR-146a was observed to affect downstream NF-*κ*B expression and, finally, inhibit inflammation [22, 23]. In contrast, miR-155 was regarded as a positive regulator in both cellular and humoral immune responses in some studies. miR-155-deficient mice failed to secrete class-switched immunoglobulins [24] and exhibited diminished production of Th17 cells [25]. Expression profiling of miRNA has been carried out in human and animal panuveitis [26, 27]. The dynamic changes of miRNAs emerge long before disease onset [27] and are proposed to contribute to NF-*κ*B and Fas ligand activation, with ultimate photoreceptor apoptosis [26].

To our knowledge, no studies have focused on the involvement of miRNAs in either animal or human AAU. Since miRNAs regulate the NF-*κ*B pathway, detailed investigation of the dynamic expression of miRNAs might provide new insights into the pathogenesis and treatment of EAAU. Specific miRNA changes can be quantitative guidance for inflammatory activity, early predictors of disease attack, and steroid-sparing therapeutic targets. Meanwhile, as evidence regarding Th17 participation in EAAU is scarce, Th1/Th17 cytokine analysis is also important in confirmation of specific cellular immune-pathogenesis in EAAU. The present study was therefore conducted to reveal the dynamic changes of miRNAs and Th1/Th17 related cytokines in EAAU.

## 2. Materials and Methods

### 2.1. Animals

Lewis rats that were 6–8 weeks old and weighed 125–160 g were used in the experiments. All animals were treated in accordance with the ARVO statement for the Use of Animals in Ophthalmic and Vision Research.

### 2.2. Preparation of Antigen and Induction of EAAU

Melanin-associated antigen (MAA) was prepared according to the method from Broekhuyse et al.'s publication in 1991 [8]. The iris and ciliary bodies were harvested from fresh bovine eyes. The tissue was homogenized and then filtered through a wire mesh to remove all the connective tissue and cellular debris. Next, the homogenate was centrifuged at 1.2 × 10^5^ g at 4°C for 15 minutes. The centrifuged homogenate was then washed once with phosphate buffered saline (PBS) at pH 7.4. The resulting pellet was resuspended in 2% sodium dodecyl sulfate (Bio-Rad, Richmond, CA, USA) and incubated at 70°C for 10 minutes. The pellet was washed three times with water after centrifugation. These insoluble antigens were subsequently dried and stored at −20°C.

In order to induce EAAU, the Lewis rats simultaneously received two injections of different MAA preparations. (1) MAA was suspended in PBS and 1 : 1 emulsified in complete Freund's adjuvant (Sigma Aldrich, St. Louis, MO, USA). The suspension (0.05 mL) was injected into the left hind footpad of the rats. (2) MAA was emulsified with 1 *μ*g purified* Bordetella pertussis* toxin (List Labs, Campbell, CA, USA) and injected intraperitoneally in a total volume of 0.05 mL. In the control group, 0.05 mL of PBS was simultaneously injected into the left hind footpad and intraperitoneally in the rats.

### 2.3. Clinical Examinations

Biomicroscopy examinations were performed daily. The disease severity was graded from 0 to 4: 0: normal, without any anterior chamber cells or iris changes; 1: slight iris vessel dilation and some anterior chamber cells; 2: iris hyperemia, with some limitation in pupil dilation; 3: miotic, hyperemic, irregular, and slightly damaged iris, with considerable flare and cells (especially when accumulated near the iris); and 4: seriously damaged and hyperemic iris, with a miotic pupil filled with protein, and cloudy, gel-like aqueous humor (AqH).

### 2.4. Tissue Preparations

Three Lewis rats each in the study and control groups were sacrificed on days 0, 7, 10, 15, and 25 after immunization. Both eyes were harvested at each time point. The eyes were enucleated, and then the iris and ciliary bodies were carefully isolated under an operating microscope. The popliteal lymph nodes were also harvested.

### 2.5. Quantification of Leukocytes in Aqueous Humor

Immediately after sacrificing the animals and before dissection of ocular tissues at each time point, the AqH was obtained using a 30-gauge needle (2 *μ*L). The AqH was then collected in silicone-treated microcentrifuge tubes (Fisher Scientific, Pittsburgh, PA, USA) and stained with 0.4% trypan blue. The numbers of leukocytes were counted under phase contrast microscopy.

### 2.6. RNA Extraction and Microarray Experiments

Total RNA was isolated from iris, ciliary bodies, and popliteal lymph nodes with Trizol reagent (Life Technologies, Gaithersburg, MD, USA). The samples were labeled using the miRCURY LNA microRNA Hi-Power Labeling Kit, Hy3/Hy5, and hybridized on the miRCURY LNA microRNA Array (7th Gen, Exiqon, Vedbæk, Denmark) in accordance with the manufacturer's instructions. Image analysis was then performed to quantify the signals on the array. The mean ± standard deviation was calculated for each group, and miRNA signals were all transformed to logarithm base 2 for further statistical analysis.

### 2.7. Quantitative Measurement of MicroRNAs, Cytokines, and Corresponding mRNA Levels

Preliminary comparison was performed between samples from three studies (14 days after induction) and three control Lewis rats. The miRNA profiling first identified a subset of 138 miRNAs with the absolute value of the log fold changes larger than 1. From these miRNAs, a thorough literature review was performed on the 30 most differentially expressed miRNAs. Finally, eight of the most relevant miRNAs were selected in accordance with the following principles: (1) significant differential expression between study and control samples was noted (log fold changes ≥2, either positively or negatively); (2) involvement in the NF-*κ*B pathway has been reported; and (3) potential ways that the miRNA contributes to uveitis have been addressed in the literature. The eight miRNAs studied were miR-155b-5p, miR-21-5p, miR-146a-5p, miR-9-3p, miR-147b, miRNA-183-5p, miRNA-182-5p, and mi-RNA-223-3p. Normalization was performed with snoRNA202 for the miRNAs. Quantitative real-time polymerase chain reaction was performed in triplicate.

Further, the cytokines of interest were interferon- (IFN-) *γ*, IL-17, IL-12A, IL-1*β*, IL-6, and IL-10. The relative mRNA expression levels of these six cytokines were studied after total RNA was extracted from the iris, ciliary bodies, and popliteal lymph nodes of the rats, as mentioned above. IL-12 and IFN-*γ* have long been recognized as Th1 key cytokines [28], while IL-1 and IL-17 are regarded as Th17 signature cytokines [29]. In order to elucidate how Th1 and Th17 are involved in EAAU and to further confirm the results obtained with mRNA, we measured the levels of IFN-*γ*, IL-17, IL-12A, and IL-1*β* in the AqH at days 0, 7, 10, 15, and 25 after immunization using a sandwich enzyme-linked immunosorbent assay (ELISA) kit (R&D Systems, Minneapolis, MN, USA) according to the manufacturer's instructions. The ELISA was repeated twice, and the samples were diluted up to a total volume of 50 *μ*L before testing. The optical density was determined at A_450_ (absorbance at 450 nm) with a microplate reader (Bio-Rad, Hercules, California, USA), and the cytokine concentration was determined from standard curves using recombinant standards supplied by the manufacturer.

### 2.8. Histopathological Proof by* In Situ* Hybridization for miRNAs

Enucleated eyes from Lewis rats were embedded in paraffin and cut into 2 *μ*m sections.* In situ* hybridization was then performed following the standard protocols provided by the manufacturer (Exiqon). The slides were prehybridized in a solution of 50% formaldehyde, 0.1% Tween, 5x SSC buffer, 9.2 mM citric acid, 50 *μ*g/mL heparin, and 500 *μ*g/mL yeast RNA. The slides were hybridized overnight in a humidified chamber at 57°C, with 20 nM of digoxigenin-labeled probe per slide. Oligonucleotide probes specific for the two selected miRNAs (miR-146a-5p and miR-182-5p) were labeled at the 5′ end with digoxigenin. Antidigoxigenin antibodies were used to detect the hybridized probes. After three washings at 57°C, the sections were stained with solutions (Kernechtrot, Muto Chemical, Tokyo, Japan).

### 2.9. Statistical Analysis

The levels of cytokine and miRNA expression and the clinical severity grading of EAAU were analyzed with the Kruskal-Wallis test. Comparisons between the study and control groups were performed with the Mann-Whitney *U* test. Continuous variables are presented as the mean ± standard deviation. *P* values < 0.05 were considered statistically significant.

## 3. Results

### 3.1. Clinical Activity Scores and Leukocyte Quantification in Aqueous Humor following Induction of EAAU

**Figure 1:**
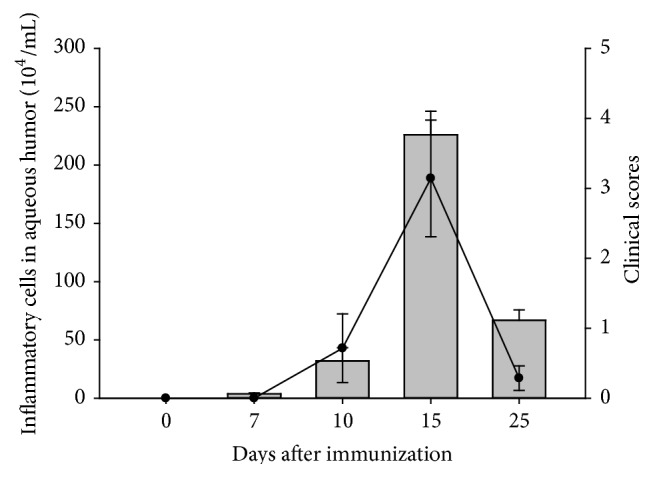
Clinical scores and inflammatory cells in aqueous humor following induction of EAAU. The concentrations of the inflammatory cells are shown in the bar chart, while the clinical scores are represented by the line graph. EAAU: experimental autoimmune anterior uveitis.

The clinical signs of EAAU and leukocyte infiltration were noted from day 7 after MAA induction, with both peaking at day 15 and decreasing at day 25 after immunization. The dynamic changes of AqH leukocyte quantification and clinical scores are shown in Figure 1, and both reached statistical significance (*P* = 0.0001 and *P* = 0.0472, resp.).

### 3.2. Relative miRNA Expression Levels in Iris and Ciliary Bodies following EAAU Induction

**Figure 2:**
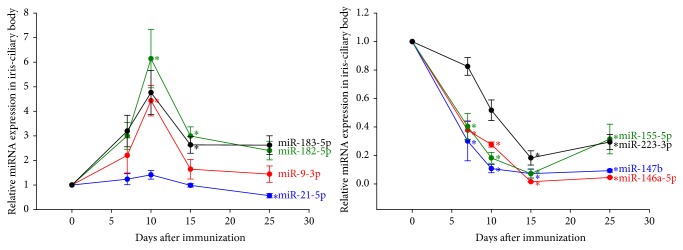
Relative expression levels of microRNAs in iris/ciliary bodies. ^*∗*^Significant differential expression among study and control eyes (*P* < 0.05).

**Figure 3:**
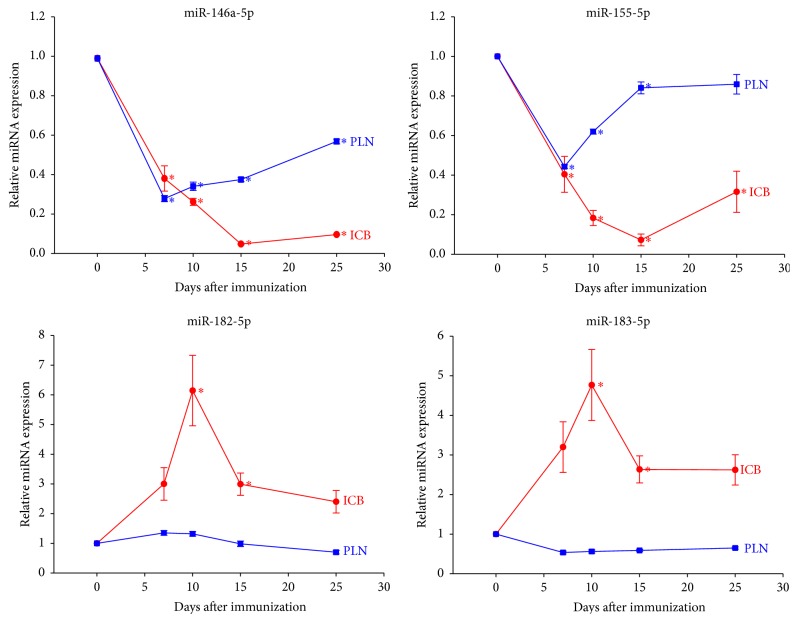
Respective dynamic expression levels of microRNAs in iris/ciliary bodies and popliteal lymph nodes. ^*∗*^Significant differential expression among study and control eyes (*P* < 0.05). ICB: iris/ciliary body tissue; PLN: popliteal lymph nodes. miR-146a-5p and miR-155-5p: downregulation of miRNA in PLN reached its lowest point at day 7 after immunization, while that in ICB reached its lowest point at day 15 after immunization. miR-182-5p and miR-183-5p: upregulation of miRNA in ICB peaked at day 7 after immunization, while dynamic changes of miRNA expression in PLN did not reach significant difference among the study and control groups at any specific time point.

**Figure 4:**
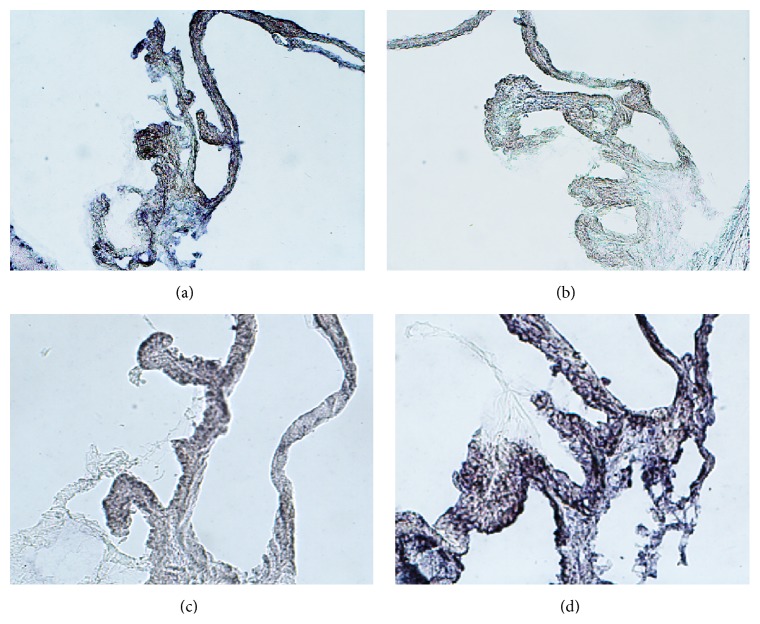
*In situ* hybridization of microRNAs in iris/ciliary body tissue. (a) + (b) miR-146-5p at day 15 after immunization: note the decreased purple dots, which reflect the lower expression in the study eye (b) than in the control eye (a). (c) + (d) miR-182-5p at day 10 after immunization: note the increased purple dots, which reflect the higher expression in the study eye (d) than in the control eye (c).

The complete dynamic profiles of the levels of each miRNA are summarized in Table 1 and Figure 2. Generally, the expression of miR-9-3p, miR-182-5p, and miR-183-5p tended to increase from day 7 after immunization onwards and peaked at day 10 after immunization. Meanwhile, miR-146a-5p, miR-147b, and miR-155-5p were significantly downregulated from day 7 and were repressed along the disease course, reaching the lowest level of expression at day 15. The expression levels of miR-146a-5p, miR-155-5p, miR-182-5p, and miR-183-5p in iris/ciliary bodies and popliteal lymph nodes are shown in Figure 3. The levels of miR-146a-5p and miR-155-5p in the popliteal lymph nodes reached their lowest point earlier, on day 7, while those in the iris/ciliary body tissue kept decreasing until day 15 after immunization. No significant difference was noted in terms of miR-182-5p and miR-183-5p in the popliteal lymph nodes over the course of the disease.* In situ* hybridization in the iris/ciliary body tissue over the 14 days following disease induction (Figure 4) confirmed the reduced expression of miR-146a-5p and enhanced expression of miR-182-5p in the eyes examined.

### 3.3. Cytokine Concentration in the Aqueous Humor and Corresponding mRNA Expression in Iris and Ciliary Bodies following EAAU Induction

**Figure 5:**
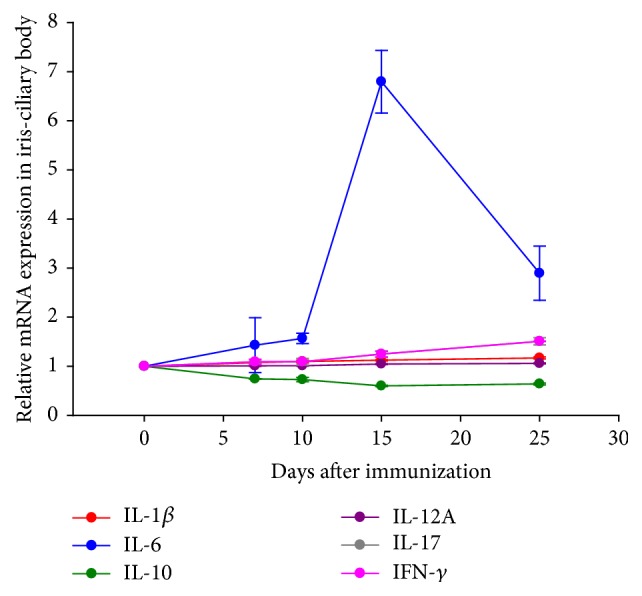
Summary of relative expression levels of mRNA of the target cytokines in iris/ciliary bodies. Because of the space limit, IL-17 and IL-12A coincide in the graph.

A summary of the relative mRNA expression levels in iris/ciliary bodies is shown in Figure 5. Among them, IL-6 showed the most obvious increase in expression, with 6.89 ± 0.28-fold changes at day 15 after immunization (*P* = 0.002), while IL-10 exhibited significantly repressed expression, with 0.63 ± 0.01-fold changes at day 15 after immunization (*P* = 0.001). The peak/trough fold changes of mRNA expression of the other four cytokines were IFN*γ*: 1.68 ± 0.03 at day 15 after immunization (*P* = 0.002), IL-12A: 1.53 ± 0.03 at day 25 after immunization (*P* = 0.004), IL-17A: 1.52 ± 0.04 at day 25 after immunization (*P* = 0.007), and IL-1*β*: 1.30 ± 0.05 at day 25 after immunization (*P* = 0.025). In order to elucidate the involvement of the Th1 and Th17 lineages and to confirm the mRNA findings, the concentrations of IFN*γ*, IL-12A, IL-17A, and IL-1*β* were analyzed with ELISA.

**Figure 6:**
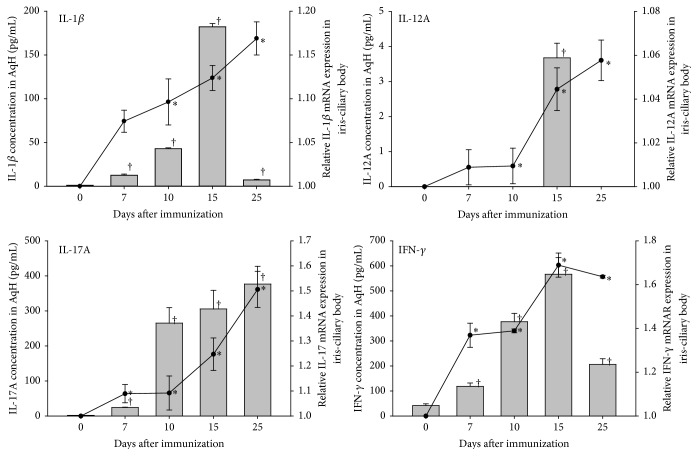
Cytokine concentrations in the aqueous humor and corresponding mRNA expression in iris/ciliary bodies following induction of EAAU. Cytokine concentrations are shown in the bar charts, while the relative mRNA expression is represented by the line graphs; ^*∗*^cytokine concentration with significant difference (*P* < 0.05) among the study and control groups; ^†^cytokine-related mRNA expression with significant difference (*P* < 0.05) among the study and control groups.

Detailed mRNA expression and aqueous concentration of each target cytokines in the disease course are shown in Figure 6. The concentration of IL-1*β* significantly increased from day 7 after immunization, peaking at day 15 and falling at day 25 after EAAU induction. IL-1*β* mRNA showed a continuously increasing trend from the beginning of the disease induction. IL-12A concentration dramatically increased at day 15 following disease induction, and decreasing soon after. The mRNA expression of IL-12A kept increasing even after the reduction of IL-12A concentration. Further, IL-17A steadily increased in concentration from day 7 after immunization, with a corresponding trend in mRNA expression. IFN-*γ* concentration and mRNA expression simultaneously increased, peaking at day 15 and falling at day 25 after EAAU induction. Overall, the dynamic changes in the concentration of each cytokine were consistent with those of the corresponding mRNAs.

## 4. Discussion

In the current study, the differential expressions of miRNAs were mostly evident 7–10 days after disease induction, with sequential cytokine changes 10–15 days after immunization. Elevation of clinical scores and corresponding leukocyte infiltration in the AqH peaked at day 15 after immunization, which was in agreement with the results in previous literature [11, 30, 31]. This time sequence suggests that regardless of proinflammatory (upregulated) or inhibitory (downregulated) roles of individual miRNAs, immunological activation occurs days before clinical presentation, and that miRNAs may be the upstream molecular driver. The earlier changes of miR-146a-5p and miR-155-5p in the popliteal lymph nodes than in the iris/ciliary bodies also demonstrate the immunoanatomical fact that antigen presentation with T cell maturation has already taken place in the closest draining lymph node.

Clinical inflammation ensues through the sequential activation of both the Th1 and Th17 lineages, as proven by the cytokine profiling. Previous studies have addressed the involvement of innate immunity early in the course of the disease, with late involvement of adaptive immunity in EAAU [8, 32]. Molecular evidence has further disclosed the involvement of NF-*κ*B in the induction of uveitis [33, 34]. Taken together with the detailed pathway of NF-*κ*B activation by upstream miRNAs in toll-like receptor signaling [35] and the role of miRNAs in T cell clonal expansion/polarization [36], the miRNA signatures in the current study provide insight into the immunopathogenesis of EAAU. Among the miRNAs studied, miR-146a-5p, miR-155-5p, miR-182-5p, and miR-183-5p are four particularly crucial miRNAs for the following reasons: they all show significant differential profiles in the disease course; they all regulate NF-*κ*B pathway, and miR-146a-5p and miR-155-5p are regarded as potent immunological drivers; they all have been reported in some uveitis models. Therefore, the interplay of these 4 miRNAs with NF-*κ*B deserves special attention.

The regulation of miR-146a and NF-*κ*B is bidirectional and encompasses both innate and adaptive immunity. While the miR-146a gene is transcriptionally activated in response to NF-*κ*B activation, it inhibits TRAF6 and IRAK1 and, hence, dampens NF-*κ*B expression [20, 22, 23]. Additionally, the Th1 lineage is normally suppressed by miR-146a through the targeting of STAT-1 expression and the activation of Treg cells [37]. Genetic studies on human uveitis revealed the association of the downregulated genotype of a single nucleotide polymorphism of miR-146a, rs2910164, with increased susceptibility to juvenile idiopathic arthritis [38] and microvascular leakage in pediatric uveitis [39]. The decreased expression of miR-146a in a Chinese population with Behçet's disease has also been noted [40]. In agreement with the aforementioned studies, the dramatic downregulation of miR-146a over the disease course possibly causes the NF-*κ*B activation and Th1 clonal expansion and, ultimately, the intraocular inflammation observed in this disease. As current evidence clearly delineated the regulation between miR-146a and NF-*κ*B, and the immune-inhibitory role has been consistently validated through various autoimmune diseases and uveitis, miR-146a can be a promising therapeutic target.

Previous literature has revealed the substantial involvement of miR-155 in both innate and adaptive immunity, including the inhibition of the MyD88-dependent toll-like receptor pathway [41], immunoglobulin class switching, Th17/IL-17 axis enhancement, and Th1 upregulation with Th2 downregulation [36]. While the positive contribution of the immune response in clinical and experimental arthritis [42] and multiple sclerosis [43] has been noted, miR-155 knockout mice suffered from an exaggerated autoimmune response in the lungs, indicating its role in the prevention of asthma [44]. These contradictory results reflect the fact that multiple targets of the intracellular pathways can be manipulated by miR-155. The downregulation of miR-155 may possibly contribute to EAAU emergence. Firstly, NF-*κ*B upstream factors such as MyD88 [45], TAB2 [46], IKK*ε*, and RIP1 [35], which are normally inhibited by miR-155, may be overexpressed following the downregulation of miR-155. Secondly, the Th1 lineage may be abated after the downregulation of miR-155, which subsequently results in high levels of Th17 axis expression. In accordance with our findings, miR-155 downregulation has also been noted in human subjects with active Behçet's disease [47].

MiR-182 and miR-183 belong to the same family, and unlike other miRNAs, miR-182 is one of the few dominant miRNAs that can increase more than 100-fold [48]. These miRNAs are abundant in retinal tissues and are necessary for maintaining the outer segments of adult cone photoreceptors and visual function [49]. Previous evidence has suggested the important role of miR-182 in T cell clonal expansion after the stimulation of T helper cells by IL-2 [50] and regulation of specialization of Treg cells [51]. Blockage of miR-182 led to the improvement of arthritis in an ovalbumin-induced arthritis mouse model [50]. Importantly, miR-182 was noted to be overexpressed in gliomas and directly suppressed cylindromatosis (CYLD), an NF-*κ*B-negative regulator [52]. The expression profile of the miR-182 family in panuveitis has been studied. In murine EAU or human sympathetic ophthalmia eyes, miR-182 and miR-183 were downregulated following disease induction and were speculated to be associated with retinal tissue injury [27] and Forkhead box O1 or Fas/Fas ligand system activation [26, 53]. A genetic association study in humans also revealed that subjects with the downregulated miR-182 genotype are more susceptible to Behçet's disease and Vogt-Koyanagi-Harada disease [54]. In current EAAU models sparing the retina, however, the results differed from those from previous panuveitis studies. We believe that, in the absence of retinal destruction, the cause of miR-182 family overexpression in EAAU is highly likely due to active involvement of NF-*κ*B activation and T cell recruitment. This discrepancy also exemplifies the heterogeneous pathogenesis in different types of uveitis.

Although cytokine profiling has been performed, few studies have provided consistent results and specific demonstration of Th1/Th17 relevance in the EAAU model. A previous study revealed that TNF-*α* and IFN-*γ* mRNA are upregulated over the disease course of EAAU and that inhibition of NF-*κ*B reduced the levels of these two proinflammatory cytokines, while augmenting the expression of anti-inflammatory cytokines such as IL-10 [11]. However, another research has revealed that IFN-*γ* mRNA is easily detectable in the popliteal lymph nodes but is barely measureable in iris/ciliary bodies. No changes in IL-10, IL-2, IL-4, or IL-6 mRNA levels were noted in the iris/ciliary bodies in the same EAAU study [16]. The results from the current study not only show the significant differential mRNA expression of IL-6 and IL-10 but also delineate the dynamic involvement of the Th1/Th17 related cytokines in EAAU.

IL-1*β* is regarded as a potent proinflammatory cytokine that is involved in many autoimmune diseases in humans [55, 56]. Some evidence has also suggested the indispensable role of IL-1*β* in uveitides involving the whole uveal tract, such as human Behçet's disease [57] and animal EAU [58]. The inflammatory cascades that result in TRAF6 and subsequent NF-*κ*B activation can be activated [59]. The upregulation of downstream cytokines such as IL-17 and IFN-*γ*, along with the development of Th17 cells, may further sustain intraocular inflammation [60]. In brief, the predominant participation of IL-1*β* in EAAU shown in our study reflects the multifunctional nature of IL-1*β* by promoting innate immunity and autoinflammation, inducing NF-*κ*B activation, enhancing Th17 activation, and introducing numerous downstream cytokines activation.

The Th17 lineage plays a pivotal role in autoimmune diseases [61] and is also considered to be crucial in uveitis activation [62–64]. By activating TRAF6, synergizing TNF-*α* expression, and inhibiting miR-23b, NF-*κ*B and other downstream pathways are strongly upregulated [61]. Interestingly, the contribution of immunopathogenesis by the Th17 and Th1 lineages can be quite complex. IFN-*γ*, the key cytokine of the Th1 axis enhanced by IL-12 [65], reportedly plays a negative regulatory role in dendritic cell function and T cell priming in EAU [66] and may protect eyes from autoimmune attacks promoted by the Th17/IL-17 axis [67, 68]. However, rather than mutual antagonization, single dominant Th1 or Th17 lineage was found to be sufficient to generate intraocular inflammation independently from the other axis [69]. Our results demonstrate the early elevation of both IL-1*β* and IFN-*γ*, which represent the Th17 and Th1 axes, respectively, at day 10 after immunization. Both cytokines remain active and synergistically promote further activation of IL-17A and IL-12 at day 15 after immunization, with a concurrent increase in clinical inflammation. The current results confirm that both Th1 and Th17 lineages are active in EAAU, just as in panuveitis.

**Figure 7:**
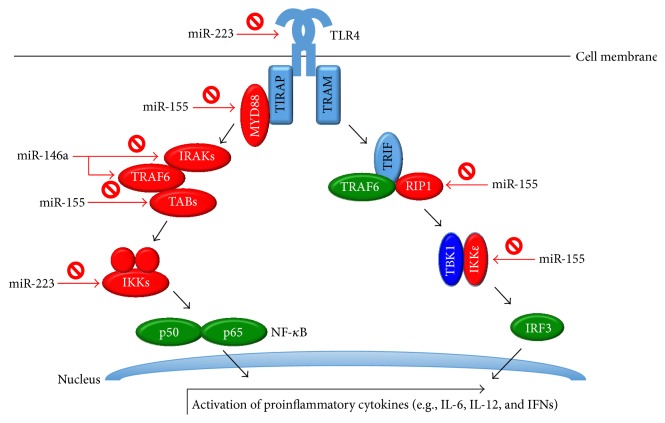
Negative regulation of toll-like receptor signaling by microRNAs. TLR: toll-like receptor; MyD88: myeloid differentiation marker 88; TRAM: TRIF-related adaptor molecule; TIRAP: TIR-associated protein; TRIF: TIR domain-containing adapter inducing; IRAK: IL-1 receptor-associated kinase; TRAF: TNF receptor-associated factor; TAB: TAK binding protein; IKK: I kappa B kinases; RIP: receptor-interacting protein; TBK: TANK-binding kinase; IRF: interferon regulatory factor; NF-*κ*B: nuclear factor kappa B; IL: interleukin; IFN: interferon; miR: microRNA. Potential target molecules regulated by miRNAs are represented in red.

**Figure 8:**
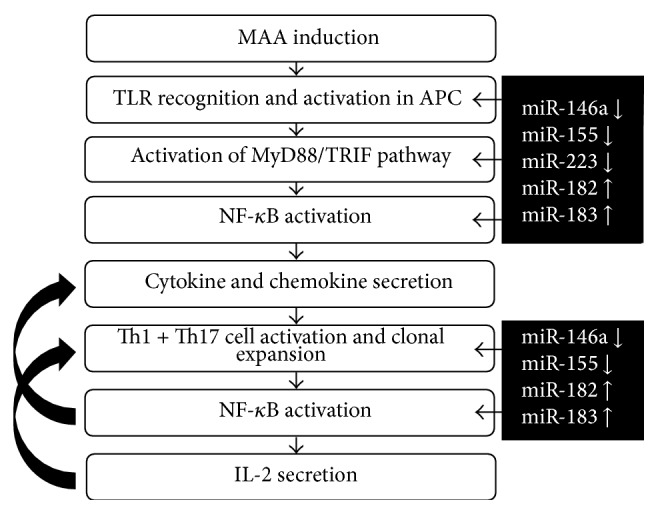
Proposed pathogenesis and respective regulations of microRNAs in EAAU. TLR: toll-like receptor; MyD88: myeloid differentiation marker 88; TRIF: TIR domain-containing adapter inducing IFN-beta; APC: antigen presenting cells; EAAU: experimental autoimmune anterior uveitis; IL: interleukin; NF-*κ*B: nuclear factor kappa B; IFN: interferon; MAA: melanin-associated antigen.

Summarizing these findings, following the recognition of MAA by toll-like receptors in antigen presenting cells, relevant miRNAs may promote the activation of NF-*κ*B and subsequent cytokine secretion (Figure 7). Further activation of T cells and polarization of the immune axis could also be influenced by miRNAs. Through multiple regulation points by miRNAs, innate and adaptive immunity manage the clinical signs and leukocyte infiltration in EAAU (Figure 8).

## 5. Conclusions

The current study provides valuable information on the dynamic changes of miRNAs and relevant Th1- and Th17-specific cytokines over the course of EAAU. miR-146a-5p, miR-155-5p, miR-147b, and miR-223-3p were downregulated, while miR-182-5p, miR-183-5p, and miR-9-3p were upregulated. Upstream changes of miRNAs contribute to NF-*κ*B activation, with further downstream activation of both Th17- and Th1-specific cytokines and effector T cells. In future, studies investigating how the miRNAs, especially miR-146a-5p, miR-155-5p, miR-182-5p, and miR-183-5p, affect each upstream NF-*κ*B signaling factor and the therapeutic effects of miRNAs in EAAU are warranted.

## Figures and Tables

**Table 1:** Detailed microRNA expression profiles over the course of EAAU.

MicroRNA	7 dpi		10 dpi		15 dpi		25 dpi	
	Fold	*P*	Fold	*P*	Fold	*P*	Fold	*P*
Upregulated miRNAs								
miR-9-3p	2.20 ± 0.53	0.15	**4.42 ± 0.78**	**0.04**	1.64 ± 0.41	0.26	1.48 ± 0.30	0.25
miR-182-5p	3.00 ± 0.55	0.07	**6.15 ± 1.19**	**0.05**	2.99 ± 0.38	0.03	2.40 ± 0.38	0.07
miR-183-5p	3.20 ± 0.64	0.08	**4.77 ± 0.90**	**0.05**	2.63 ± 0.34	0.04	2.62 ± 0.38	0.05

Downregulated miRNAs								
miR-146a-5p	0.37 ± 0.05	<0.01	0.25 ± 0.02	<0.01	**0.05 ± 0.00**	**<0.01**	0.10 ± 0.01	<0.01
miR-147b	0.31 ± 0.08	0.01	0.12 ± 0.01	<0.01	**0.07 ± 0.02**	**<0.01**	0.09 ± 0.02	<0.01
miR-155-5p	0.42 ± 0.08	0.02	0.15 ± 0.03	<0.01	**0.08 ± 0.01**	**<0.01**	0.31 ± 0.07	0.01
miR-223-3p	0.82 ± 0.09	0.18	0.51 ± 0.08	0.02	**0.18 ± 0.02**	**<0.01**	0.29 ± 0.03	<0.01

miRNA with mixed trends								
miR-21-5p	1.28 ± 0.24	0.35	1.42 ± 0.18	0.14	0.99 ± 0.07	0.86	0.56 ± 0.09	0.04

Fold: fold changes among study/control eyes, expressed in mean ± standard deviation; dpi: days after immunization; EAAU: experimental autoimmune anterior uveitis

The peak expression levels among upregulated miRNAs and the trough expression levels among downregulated miRNAs are expressed in bold.
